# A cyclin-dependent kinase inhibitor, dinaciclib in preclinical treatment models of thyroid cancer

**DOI:** 10.1371/journal.pone.0172315

**Published:** 2017-02-16

**Authors:** Shu-Fu Lin, Jen-Der Lin, Chuen Hsueh, Ting-Chao Chou, Richard J. Wong

**Affiliations:** 1 Department of Internal Medicine, Chang Gung Memorial Hospital, Chang Gung University, Taoyuan, Taiwan; 2 Department of Pathology, Chang Gung Memorial Hospital, Chang Gung University, Taoyuan, Taiwan; 3 Laboratory of Preclinical Pharmacology Core, Memorial Sloan-Kettering Cancer Center, New York, New York, United States of America; 4 Department of Surgery, Memorial Sloan-Kettering Cancer Center, New York, New York, United States of America; Columbia University, UNITED STATES

## Abstract

**Background:**

We explored the therapeutic effects of dinaciclib, a cyclin-dependent kinase (CDK) inhibitor, in the treatment of thyroid cancer.

**Materials and methods:**

Seven cell lines originating from three pathologic types of thyroid cancer (papillary, follicular and anaplastic) were studied. The cytotoxicity of dinaciclib was measured using a lactate dehydrogenase assay. The expression of proteins associated with cell cycle and apoptosis was assessed using Western blot analysis and immunofluorescence microscopy. Cell cycle distribution was measured by flow cytometry and immunofluorescence microscopy. Apoptosis and caspase-3 activity were measured by flow cytometry and fluorometric assay. Mice bearing flank anaplastic thyroid cancer (ATC) were treated with intraperitoneal injections of dinaciclib.

**Results:**

Dinaciclib inhibited thyroid cancer cell proliferation in a dose-dependent manner. Dinaciclib had a low median-effect dose (≤ 16.0 nM) to inhibit cell proliferation in seven thyroid cancer cell lines. Dinaciclib decreased CDK1, cyclin B1, and Aurora A expression, induced cell cycle arrest in the G2/M phase, and induced accumulation of prophase mitotic cells. Dinaciclib decreased Mcl-1, Bcl-x_L_ and survivin expression, activated caspase-3 and induced apoptosis. *In vivo*, the growth of ATC xenograft tumors was retarded in a dose-dependent fashion with daily dinaciclib treatment. Higher-dose dinaciclib (50 mg/kg) caused slight, but significant weight loss, which was absent with lower-dose dinaciclib (40 mg/kg) treatment.

**Conclusions:**

Dinaciclib inhibited thyroid cancer proliferation both *in vitro* and *in vivo*. These findings support dinaciclib as a potential drug for further studies in clinical trials for the treatment of patients with refractory thyroid cancer.

## Introduction

Thyroid cancer is a common endocrine malignancy with an increasing incidence in the past three decades. There are three major histologic types of thyroid cancer originate from thyroid follicular cells: papillary (PTC), follicular (FTC) and anaplastic (ATC) thyroid cancer [1]. More than 90% patients with thyroid malignancy are PTC and FTC, known as well-differentiated thyroid cancer (WDTC). Patients with WDTC usually survive for more than 10 years following treatment with surgery, radioactive iodine (RAI) and thyroid hormone suppressive or replacement therapy. However, some patients who develop metastatic and RAI-refractory WDTC have survival of < 3–5 years [2]. Two multi-kinase inhibitors, sorafenib and lenvatinib, have recently been approved by the U.S. Food and Drug Administration for the treatment of metastatic and RAI-refractory WDTC. However, the therapeutic efficacy of these agents is limited in many patients who eventually develop disease progression and require additional therapy [3,4]. ATC is a rare but fatal disease with a median survival of 6 months, and most patients die within 1 year after diagnosis [5]. Novel treatments with different therapeutic mechanisms are crucial for improving the outcomes of these patients with refractory thyroid cancer.

Unrestricted cell proliferation, one of the hallmarks of malignant tumors, is often driven by alterations in cyclin-dependent kinase (CDK) activity. CDKs are serine/threonine kinases that regulate the cell cycle by interacting with specific cell cycle regulatory cyclins. According to the classical model of cell cycle control in the mammalian cells, CDK4 and CDK6 are activated by D-type cyclins and induce cells to transition from quiescent (G0) to the first gap phase (G1). In the late G1 phase, CDK2 is activated by cyclin E, driving G1 to S phase transition. Cell cycle progression is further promoted by the CDK2-cyclin A and CDK1-cyclin A complexes. Finally, the CDK1-cyclin B1 complex is responsible for entry and progression of the mitotic phase (M) [6–8]. Altered CDKs activity is observed in many cancers [9,10]. In addition to cell cycle regulation, the CDK family has other biologic functions. For example, CDK5 is important for neural development in embryos and promotes proliferation of the medullary thyroid cancer cell line [11,12]. CDK9 phosphorylates the C-terminal domain of RNA polymerase II, promoting RNA transcription [13]. Given the important role of CDKs in the regulation of cell proliferation, inhibition of their activity may represent a promising strategy in the treatment of human malignancies, including thyroid cancer [14].

Dinaciclib is a potent and specific CDK inhibitor that represses CDK1, CDK2, CDK5 and CDK9 with 50% inhibitory concentrations in the low nanomolar range (3, 1, 1 and 4 nM, respectively) [15]. Preclinical studies have shown that dinaciclib can arrest cell cycle progression, induce apoptosis and inhibit tumor growth in multiple types of cancer [15–19]. In addition, a recent clinical trial demonstrated this drug was well-tolerated and had single agent activity against multiple myeloma, supporting its potential in treating human malignancies [20].

In this study, we evaluated the therapeutic effects of dinaciclib on seven thyroid cancer cell lines of follicular cell origin, including PTC, FTC and ATC.

## Materials and methods

### Cell lines

Seven human thyroid cancer cell lines were evaluated, including a papillary (BHP7-13), a follicular (WRO82-1), a follicular undifferentiated (FRO81-2) and four anaplastic (8305C, 8505C, KAT18, KAT4C) cancer cell lines [21–25]. With the exception of KAT4C cells, for which no DNA short tandem repeat (STR) profile is available, all cell lines were authenticated using DNA STR profiling and stored in liquid nitrogen until use. BHP7-13, WRO82-1, FRO81-2, KAT18 and KAT4C were maintained in RPMI 1640 with sodium bicarbonate (2.0 g/L). 8305C and 8505C were maintained in MEM with sodium pyruvate (1 mmol/L) and sodium bicarbonate (2.2 g/L). All media contained 10% FCS, 100,000 units/L penicillin and 100 mg/L streptomycin. All cells were maintained in a 5% CO_2_ humidified incubator at 37°C.

### Pharmacologic agents

Dinaciclib was obtained from Selleck Chemicals and was dissolved in DMSO (Sigma) to a concentration of 10 mM and stored at -80°C until further use *in vitro* experiments. For the *in vivo* studies, dinaciclib was diluted in 20% (w/v) of 2-hydroxypropyl-β-cyclodextrin (Sigma) and stored at -30°C until use.

### Antibodies

Antibodies targeting cyclin B1, Aurora A, Mcl-1, Bcl-x_L_, survivin and pro-caspase-3 were purchased from Cell Signaling Technology. CDK1 and α-tubulin antibodies were obtained from Sigma.

### Cytotoxicity assays

Cells were plated at 2 x 10^3^ (BHP7-13, FRO81-2, 8505C), 1 x 10^4^ (KAT18) or 2 x 10^4^ (WRO82-1, 8305C, KAT4C) cells per well in 24-well plates in 1 mL media. After an overnight incubation, six serial 1:1 dilutions of dinaciclib or vehicle were added at the starting dose of 100 nM over a 4-day treatment course. Cytotoxicity was determined on day 4. Culture medium was removed and cells were washed with PBS and lysed with Triton X-100 (1.35%, Sigma) to release intracellular lactate dehydrogenase (LDH), which was quantified with a Cytotox 96 kit (Promega) at 490 nM by spectrophotometry (Infinite M200 PRO, Tecan). Each experiment was performed in triplicate, and the results are shown as the percentage of surviving cells determined by comparing the LDH of each sample relative to control samples, which were considered 100% viable. Median-effect dose (Dm) on day 4 was calculated for each cell line using CompuSyn software [26,27]. Dm is the dose inhibiting cell viability by 50%.

### Cell cycle assessment

The effects of dinaciclib on cell cycle progression were evaluated. Cells were plated at 4 x 10^4^ (KAT4C) or 1 x 10^5^ cells (all other cell lines) per well in 6-well plates in 2 mL of media overnight. Dinaciclib (25 nM), a clinically relevant dose [19], or vehicle was added and incubated for 24 h, after which adherent cells were trypsinized, washed with PBS, fixed with cold 70% ethanol and incubated with RNase A (100 μg/mL; Sigma) and propidium iodide (PI, 5 μg/mL; Sigma) at 37°C for 15 min. Cell cycle distribution was assessed by DNA content detected by flow cytometry (BD FACScalibur Flow Cytometer, BD Biosciences). Each condition was performed in triplicate.

### Immunofluorescence microscopy

The effect of dinaciclib on mitotic progression was evaluated using confocal microscopy. Thyroid cancer cells were plated at 5 x 10^4^ (WRO82-1) or 1 x 10^5^ (BHP7-13, 8505C) in four-well culture slides in 1 mL of media overnight. Cells were treated with dinaciclib (25 nM) or placebo for 24 h, washed with PBS, fixed in 4% paraformaldehyde (Sigma) for 15 min at room temperature, washed with PBS, permeabilized with 0.1% Triton X-100 (10 min, room temperature), washed with PBS, incubated with 4',6-diamidino-2-phenylindole (DAPI; 0.2 μg/mL, Invitrogen) for 10 min at room temperature, washed with PBS, and covered with Vectashield mounting medium (Vector Laboratories). Images were captured with Leica TCS SP8 X confocal microscopy (Leica Microsystems). Chromosomes were examined to identify mitotic cells.

The expression of cyclin B1 and Aurora A was evaluated using immunofluorescence microscopy. Dinaciclib (25 nM) or placebo treated thyroid cancer cell samples were prepared as described above. Cells were then incubated with primary rabbit cyclin B1 antibody (1:200), rabbit Aurora A antibody (1:200) and mouse α-tubulin antibody (1:1000) at 4°C overnight, washed with PBS and incubated with secondary Alexa Fluor 633-conjugated goat anti-rabbit antibody (1:1000; Invitrogen) and Alexa Fluor 488-conjugated goat anti-mouse antibody (1:1000; Life Technologies) for 25 min at 37°C, washed with PBS, counterstained with DAPI, washed with PBS and covered with mounting medium. Images were acquired using Leica TCS SP8 X confocal microscopy.

### Western blot analysis

Cells were plated at 1 x 10^6^ cells in 100-mm Petri dishes in 10 mL of media overnight and treated with dinaciclib at 25 nM or vehicle for the indicated periods. Cell pellets were dissolved in radio-immunoprecipitation assay buffer and protease inhibitor cocktail, vortexed and clarified by centrifugation. Total protein (40 μg) was separated by electrophoresis on 6% or 12% Tris-HCl gels, transferred to polyvinylidene difluoride membranes, blocked and exposed to primary antibodies followed by a secondary antibody conjugated to horseradish peroxidase. Signals were developed using an enhanced chemiluminescence kit (PerkinElmer).

### Apoptosis assessment

Caspase-3 activity was analyzed using fluorometric assay kit (Abcam). Cells were plated at 1 x 10^6^ cells in 100-mm Petri dishes in 10 mL of media overnight. Dinaciclib (25 nM) or vehicle was added for 24 h. Adherent cells (5 x 10^5^) were collected, centrifuged, lysed using 50 μL of lysis buffer on ice for 10 min, incubated with DEVD-AFC substrate and reaction buffer at 37°C for 1.5 h. Caspase-3 activity was detected by spectrophotometry. The fluorescence intensity of the treated samples was compared with that of control samples to determine the fold-increase in caspase activity. Each condition was performed in duplicate.

Early apoptosis was measured by Annexin V-Alexa Fluor 488 and PI staining kit (Invitrogen). Cells were plated at 1 x 10^5^ cells per well in 6-well plates in 2 mL of media overnight and treated with dinaciclib (25 nM) or placebo for 24 h. Adherent cells were collected, washed with PBS and incubated with Annexin V-Alexa Fluor 488 and PI at room temperature in the dark for 15 min per the manufacturer’s protocol. Early apoptotic cells (Annexin V-positive, PI-negative) were detected by flow cytometry (BD FACScalibur Flow Cytometer, BD Biosciences). Each condition was performed in triplicate.

Sub-G1 apoptosis was detected using flow cytometry. Cells were plated at 4 x 10^4^ (KAT4C) or 1 x 10^5^ cells (all other cell lines) per well in 6-well plates in 2 mL of media overnight and treated with dinaciclib (25 nM) or vehicle. Both floating cells and trypsinized adherent cells were collected at 72 h and processed as described above for cell cycle assessment. Each experiment was performed in triplicate and the proportion of apoptotic sub-G1 cells was determined by measuring the DNA content using flow cytometry.

### Flank xenograft tumor therapy

Eight-week-old athymic female nude mice from the National Laboratory Animal Center, Taiwan, were anesthetized with an intraperitoneal injection of 2% 2,2,2-Tribromoethanol (200 μl/mouse; Sigma) before implantation of thyroid cancer cells. 8505C flank tumors were established by injecting 1 x 10^6^ cells in 100 μL of ECM gel (Sigma) into the subcutaneous flanks of nude mice. When 8505C tumors reached 5.0 mm in mean diameter, mice received daily intraperitoneal injections of vehicle (*n* = 13), lower-dose dinaciclib (40 mg/kg, *n* = 13) or higher-dose dinaciclib (50 mg/kg, *n* = 7). These doses were chosen based on a previous report [15]. Tumor dimensions were serially measured with electronic calipers, and the volumes were calculated by the following formula: a x b^2^ x 0.4, where a represents the largest diameter and b is the perpendicular diameter. The body weight of each animal was followed as a marker of toxicity.

Tumor levels of CDK1, cyclin B1, Aurora A, Mcl-1, Bcl-x_L_, survivin and pro-caspase-3 were evaluated in mice treated with daily intraperitoneal injections of lower-dose dinaciclib (40 mg/kg) by Western blot analysis. At indicated periods, animals were euthanized with carbon dioxide, and the tumors were harvested, mixed with protein extraction buffer (GE Healthcare), homogenized and sonicated on ice. After centrifugation, clarified supernatants were aliquoted and stored at -80°C for Western blotting.

This study was performed in accordance with the recommendations in the Guide for the Care and Use of Laboratory Animals of the Chang Gung Memorial Hospital, and the protocol was approved by the Committee of Laboratory Animal Center at the Chang Gung Memorial Hospital, Linkou (permission No: 2013121401). Housing and care of mice were provided by the Laboratory Animal Center, Chang Gung Memorial Hospital, Linkou. Animals were given *ad libitum* access to food and water. The physical condition of mice was monitored by our animal care personnel on a daily basis. In addition, the investigators checked the conditions of the mice at least once a week before treatment, and daily during the treatment period. The humane endpoints were a tumor diameter ≥ 2.0 cm, significant weight loss (20% of pre-experiment body weight), weight loss to a final weight of 16 g, very slow breathing rate, shallow or labored breathing pattern, decreased activity, poor response to handling, absence of grooming, social isolation, hunched posture, shivering, and muscle atrophy. The method of euthanasia was CO_2_ exposure for 10 min, at a 20% fill rate of cage volume/min.

### Statistical analyses

Comparisons were performed when appropriate using two-sided Student’s *t* tests (Excel, Microsoft). *P* < 0.05 was considered statistically significant. Results were expressed as mean ± SE.

## Results

### Dinaciclib induced cytotoxicity in thyroid cancer cell lines

**Fig 1:**
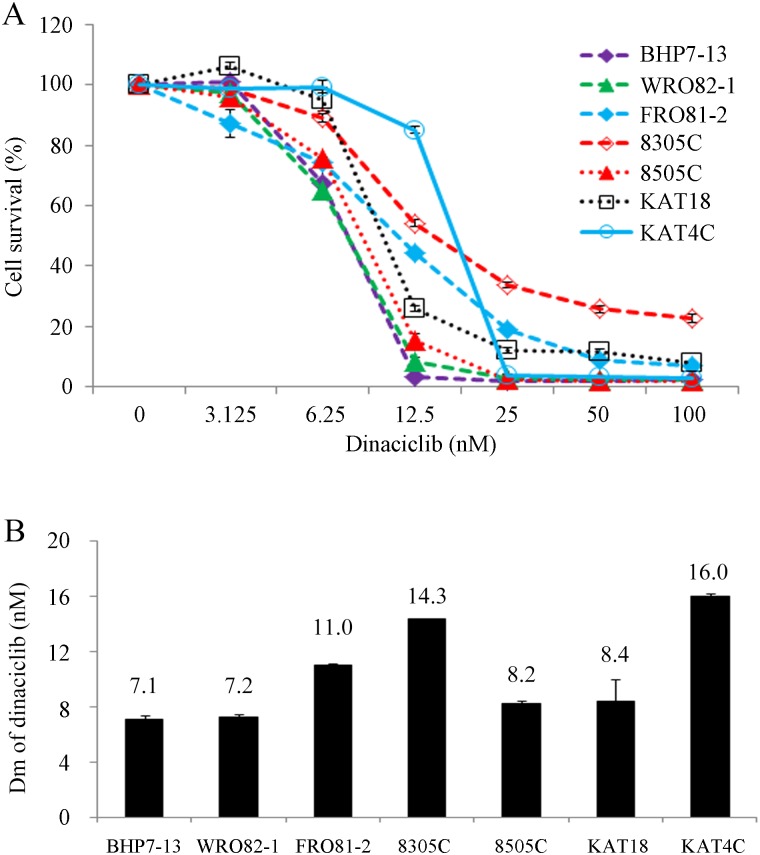
Dinaciclib induces cytotoxicity in thyroid cancer cells. (A) Cytotoxicity was evaluated in cells treated with a series of six 1:1 dilutions of dinaciclib. Dose-response curves were obtained on day 4 using a LDH assay. (B) Median-effect dose (Dm) of dinaciclib on day 4 was calculated for each cell line using CompuSyn software.

Dinaciclib repressed cell proliferation in all thyroid cancer lines in a dose-dependent manner (Fig 1A). A clinically achievable dose (25 nM) inhibited at least 66% of cell growth in all cell lines by day 4. At 100 nM, dinaciclib arrested > 97% cell growth in the WDTC lines and > 77% in the ATC lines. The potency of cytotoxicity of dinaciclib in various thyroid cancer cell line was determined by the median-effect plot of the mass-action law [26] using the CompuSyn software [27]. The median-effect doses were determined following each set of dose-effect data entries on day 4 (Fig 1B). The well-differentiated papillary and follicular thyroid cancer cell lines (BHP7-13 and WRO82-1) were the most sensitive (Dm = 7.1 ± 0.3 nM and 7.2 ± 0.2 nM, respectively). Two ATC cell lines (KAT4C and 8305C) were the most resistant (Dm = 16.0 ± 0.2 nM and 14.3 ± 0.1 nM, respectively).

### Effects of dinaciclib on the cell cycle

**Fig 2:**
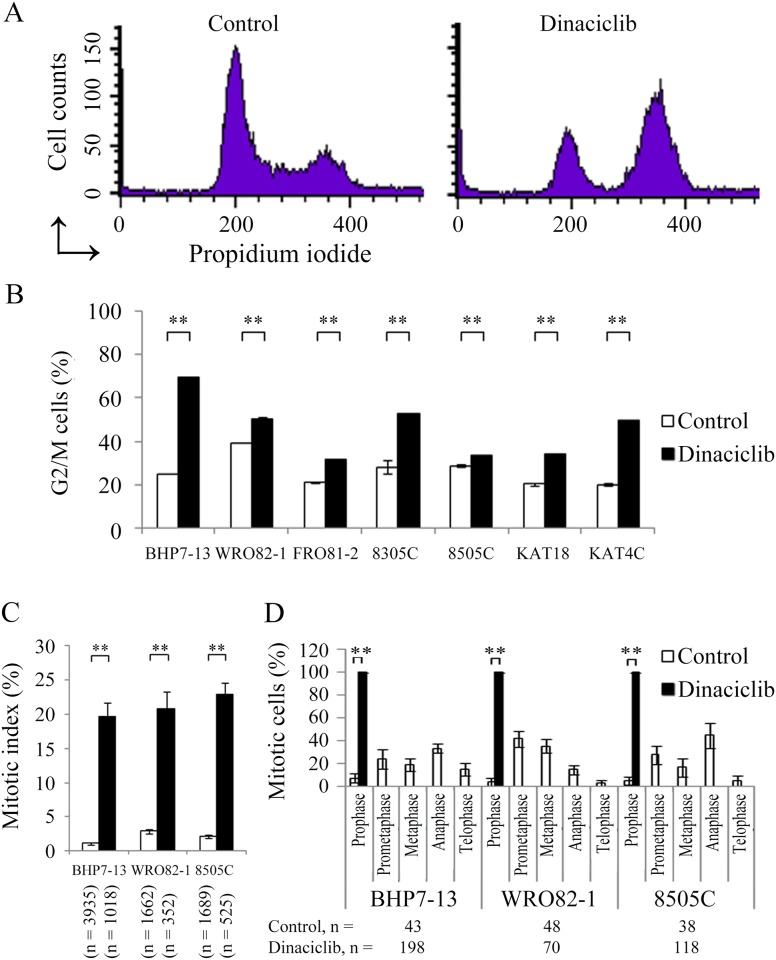
Dinaciclib accumulates cells in G2/M phase and inhibits mitotic progression in prophase. (A) Cell cycle analysis was undertaken by evaluating the DNA content using flow cytometry in BHP7-13 cells treated with placebo or dinaciclib (25 nM) for 24 h. (B) Statistical analyses revealed that dinaciclib (25 nM) significantly arrested cells in the G2/M phase at 24 h in all seven thyroid cancer cell lines. (C) The percentage of thyroid cancer cells in mitosis was assessed after treatment with placebo or dinaciclib (25 nM) for 24 h. Cells were stained with DAPI and chromosome features were evaluated using immunofluorescence confocal microscopy. Mitotic index was assessed with a minimum of 352 cells counted from at least ten different fields for each condition. Dinaciclib significantly increased the proportion of cells in mitosis in three thyroid cancer cell lines. (D) The distribution of cells in mitosis was determined by counting a minimum of 38 mitotic cells from ten different fields by confocal microscopy for each condition. Quantification analyses revealed 100% of mitotic cells were in prophase by the treatment of dinaciclib (25 nM) for 24 h. ** *P* < 0.005 compared with vehicle-treated cells.

The effect of dinaciclib (25 nM for 24 h) on cell cycle distribution was evaluated. One representative thyroid cancer cell line, BHP7-13, revealed that dinaciclib arrested cell cycle progression in G2/M phase (Fig 2A). Similar cell cycle data were analyzed for all seven cell lines (Fig 2B). Compared with control cells, dinaciclib significantly induced cell accumulation in G2/M phase in BHP7-13 (69.3 ± 0.4% and 25.2 ± 0.1%, *P* < 0.001), WRO82-1 (50.1 ± 0.8% and 39.5 ± 0.3%, *P* < 0.001), FRO81-2 (31.9 ± 0.2% and 21.3 ± 0.3%, *P* < 0.001), 8305C (52.7 ± 0.1% and 28.3 ± 3.2%, *P* = 0.002), 8505C (33.4 ± 0.3% and 28.9 ± 0.7%, *P* = 0.004), KAT18 (34.0 ± 0.3% and 20.5 ± 0.6%, *P* < 0.001) and KAT4C (49.5 ± 0.3% and 20.3 ± 0.5%, *P* < 0.001), demonstrating induction of G2/M arrest.

The ability of dinaciclib to accumulate cells in mitotic phase was determined using confocal fluorescence microscope (S1 Fig). Mitotic cells were identified and mitotic index was calculated for three thyroid cancer cell lines representing papillary (BHP7-13), follicular (WRO82-1) and anaplastic (8505C) thyroid cancer (Fig 2C). Compared with control cells, dinaciclib (25 nM) treatment for 24 h significantly increased the percentage of mitotic cells in BHP7-13 (19.7 ± 2.0% and 1.2 ± 0.2%, *P* < 0.001), WRO82-1 (20.8 ± 2.5% and 2.9 ± 0.3%, *P* < 0.001) and 8505C (22.9 ± 1.7% and 2.2 ± 0.2%, *P* < 0.001), demonstrating that dinaciclib arrested cells in mitosis.

The distribution of cells in mitosis was evaluated (Fig 2D). Compared with control treatment, dinaciclib significantly increased the percentage of prophase cells in BHP7-13 (100 ± 0% and 7.5 ± 4.0%, *P* < 0.001), WRO82-1 (100 ± 0% and 4.5 ± 3.4%, *P* < 0.001) and 8505C (100 ± 0% and 5.3 ± 3.7%, *P* < 0.001), revealing mitotic arrest was in the first phase of mitosis.

Viable cells were less than 4% when compared with control cells by the treatment of dinaciclib at 25 nM on day 4 in 4 of 7 cell lines. The limited numbers of viable cells precluded further analyses of cell cycle on day 4 in all 7 cell lines.

### Dinaciclib modulates the expression of CDK1, cyclin B1 and Aurora A

**Fig 3:**
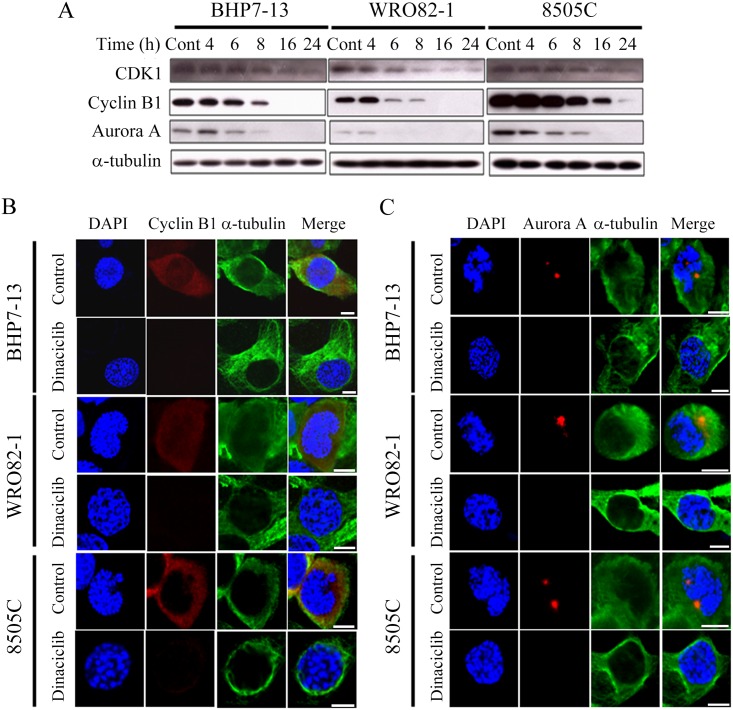
Dinaciclib decreases the levels of CDK1, cyclin B1 and Aurora A in thyroid cancer cell lines. (A) The expression of these cell-cycle associated proteins was evaluated by Western blotting in BHP7-13, WRO82-1 and 8505C cells treated with dinaciclib (25 nM) or placebo for the indicated periods. (B) Cells were treated with dinaciclib (25 nM) or placebo for 24 h and stained with fluorescent antibodies against DAPI (blue), cyclin B1 (red) and α-tubulin (green). Cyclin B1 level was significantly reduced after treatment of dinaciclib in prophase cells of BHP7-13, WRO82-1 and 8505C. (C) Cells were treated with dinaciclib (25 nM) or placebo for 24 h and stained with fluorescent antibodies against DAPI (blue), Aurora A (red) and α-tubulin (green). Aurora A level was significantly reduced after treatment of dinaciclib in BHP7-13, WRO82-1 and 8505C cells in prophase. Scale bar, 10 μm.

CDK1, cyclin B1 and Aurora A are essential proteins for G2/M transition and mitotic progression [28–30]. The effects of dinaciclib (25 nM) on the expression of these proteins were evaluated in three cell lines (Fig 3A). The intensity of the bands was quantified using the Molecular Imager VersaDoc MP 4000 system (Bio-Rad). The ratios of CDK1, cyclin B1, and Aurora A to α-tubulin in each cell line were calculated. Relative expression was calculated using the control value as reference (S2 Fig). CDK1 level was decreased by 8 h in BHP7-13 and WRO82-1 and by 24 h in 8505C. Cyclin B1 expression was decreased by 6 h in WRO82-1, by 8 h in BHP7-13, and by 24 h in 8505C. Aurora A was decreased by 6 h in BHP7-13, WRO82-1, and 8505C. These results demonstrate that 24-h dinaciclib treatment reduces CDK1, cyclin B1, and Aurora A expression in thyroid cancer cell lines. Confocal fluorescence microscopy demonstrated that dinaciclib significantly decreased cyclin B1 (Fig 3B) and Aurora A (Fig 3C) expression in prophase cells of BHP7-13, WRO82-1 and 8505C. These findings are consistent with prior results showing that dinaciclib was able to decrease CDK1 and cyclin B1 [16,17].

### Effects of dinaciclib on apoptosis

**Fig 4:**
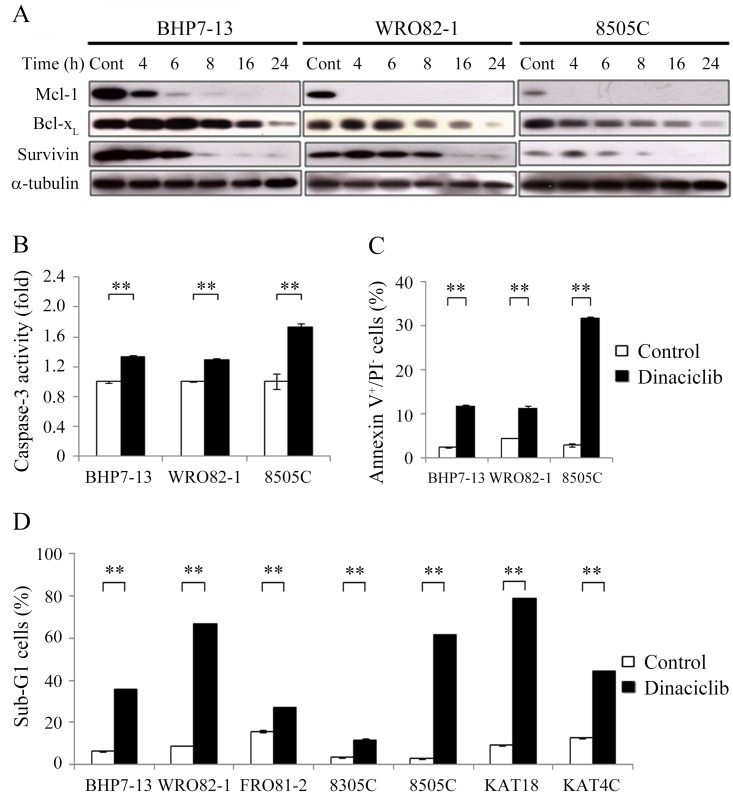
Dinaciclib decreases Mcl-1, Bcl-x_L_ and survivin levels, activates caspase-3 and induces apoptosis in thyroid cancer cells. (A) Western blot analysis was performed in cells treated with dinaciclib (25 nM) or vehicle for the indicated time. The levels of Mcl-1, Bcl-x_L_ and survivin were evaluated in BHP7-13, WRO82-1 and 8505C cells. (B) Caspase-3 activity was detected using fluorometric assay kit in cells treated with dinaciclib (25 nM) or vehicle for 24 h. (C) Statistical analyses of early apoptotic cells determined by flow cytometry to detect Annexin V-positive and PI-negative staining showed dinaciclib significantly induced early apoptosis at 24 h in BHP7-13, WRO82-1 and 8505C cells. (D) Sub-G1 apoptosis was detected by measuring the DNA content using flow cytometry in cells treated with dinaciclib (25 nM) or vehicle for 72 h. Dinaciclib increased the proportions of sub-G1 cells in all seven thyroid cancer cell lines. ** *P* < 0.005 compared with vehicle-treated cells.

Dinaciclib alters the expression of apoptosis proteins, including Mcl-1, Bcl-x_L_ and survivin, and induces apoptosis in ovarian cancer and osteosarcoma cells [16,17]. We evaluated the effects of dinaciclib on the expression of Mcl-1, Bcl-x_L_ and survivin in BHP7-13, WRO82-1 and 8505C cells (Fig 4A). Band densities were imaged and quantified. The ratios of Mcl-1, Bcl-x_L_, and survivin to α-tubulin in each cell line were calculated. Relative expression was calculated using the control value as reference (S3 Fig). Dinaciclib (25 nM) significantly decreased Mcl-1 levels by 4 h in BHP7-13, WRO82-1 and 8505C. The inhibitory effects persisted for 24 h. Bcl-x_L_ was decreased by 8 h in WRO82-1 and by 16 h in BHP7-13 and 8505C. Survivin was decreased by 8 h in BHP7-13 and 8505C and by 16 h in WRO82-1. Thus, dinaciclib decreased the levels of anti-apoptotic proteins in thyroid cancer cell lines.

The decreased levels of anti-apoptotic proteins may lead to activation of executioner caspase-3 [31,32]. The effects of dinaciclib (25 nM) on caspase-3 activity were determined using a fluorometric assay at 24 h in BHP7-13, WRO82-1 and 8505C cells (Fig 4B). Dinaciclib significantly increased caspase-3 activity when compared with control in BHP7-13 (1.338 ± 0.011-fold and 1.000 ± 0.013-fold, *P* = 0.003), WRO82-1 (1.295 ± 0.015-fold and 1.000 ± 0.011-fold, *P* = 0.004) and 8505C (1.737 ± 0.042-fold and 1.000 ± 0.107-fold, *P* = 0.023).

Activation of caspase-3 may result in apoptotic cell death. The effect of dinaciclib (25 nM) on early apoptosis was evaluated using Annexin V-Alexa Fluor 488 and PI staining at 24 h in three thyroid cancer cell lines. Statistical analyses show dinaciclib significantly increased early apoptotic cells when compared with control treatment in BHP7-13 (11.8 ± 0.4% and 2.4 ± 0.1%, *P* < 0.001), WRO82-1 (11.1 ± 0.7% and 4.5 ± 0.1%, *P* < 0.001) and 8505C (31.6 ± 0.4% and 2.9 ± 0.3%, *P* < 0.001) (Fig 4C). To confirm that dinaciclib can induce apoptosis in all thyroid cancer cell lines, seven thyroid cancer lines were exposed to dinaciclib (25 nM) or placebo for 72 h, and the proportions of sub-G1 cells were calculated (Fig 4D). Dinaciclib significantly increased the proportion of sub-G1 cells when compared with control in BHP7-13 (35.9 ± 0.2% and 6.5 ± 0.3%, *P* < 0.001), WRO82-1 (66.6 ± 0.5% and 9.0 ± 0.1%, *P* < 0.001), FRO81-2 (27.2 ± 0.3% and 15.8 ± 0.6%, *P* < 0.001), 8305C (11.7 ± 0.6% and 3.6 ± 0.1%, *P* < 0.001), 8505C (61.4 ± 0.2% and 2.9 ± 0.1%, *P* < 0.001), KAT18 (78.8 ± 0.3% and 9.3 ± 0.3%, *P* < 0.001) and KAT4C (44.2 ± 0.5% and 12.7 ± 0.5%, *P* < 0.001), demonstrating induction of apoptosis.

These cell cycle and apoptosis data reveal the cytotoxicity of dinaciclib was mediated through both cell cycle arrest and apoptosis in thyroid cancer cell lines.

### Dinaciclib therapy of murine flank tumors

**Fig 5:**
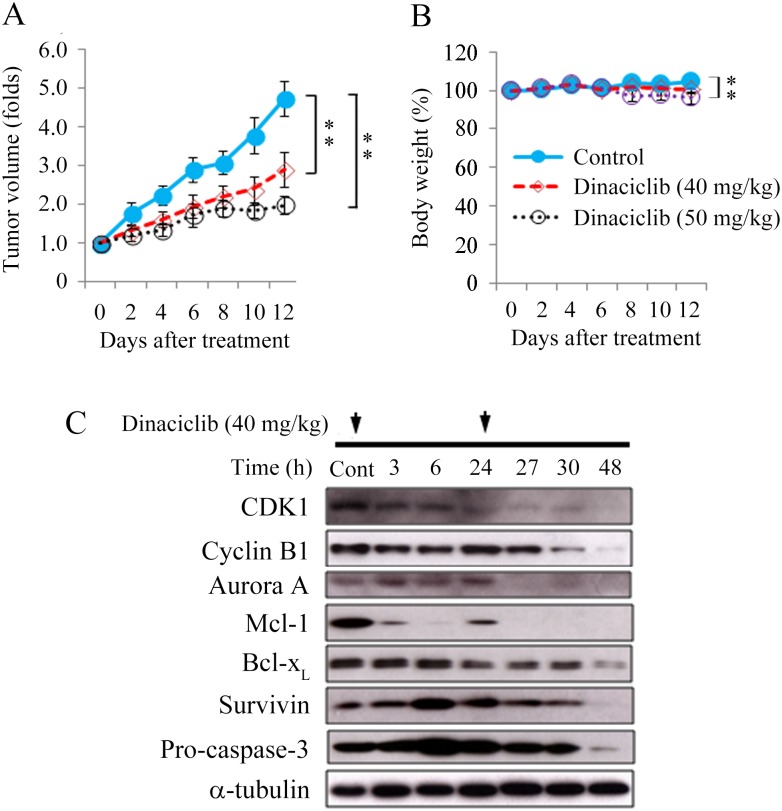
Dinaciclib inhibits subcutaneous xenograft growth of anaplastic thyroid cancer. (A) The therapeutic effects of dinaciclib were evaluated in mice bearing 8505C flank tumors. Daily intraperitoneal injections of lower-dose (40 mg/kg) and higher-dose (50 mg/kg) dinaciclib significantly repressed 8505C tumor growth after 6 and 4 days when compared with control mice, respectively. (B) Daily treatment of lower-dose dinaciclib did not meaningfully induce weight loss when compared with control mice during the study period. However, higher-dose dinaciclib induced significant weight loss between days 8 and 12. (C) The molecular effects of daily lower-dose dinaciclib (40 mg/kg) treatment were evaluated in 8505C tumors using Western blot analysis. ** *P* < 0.005 compared with vehicle-treated mice on day 12.

Nude mice bearing flank xenografts of 8505C were used to study the therapeutic efficacy and safety of dinaciclib *in vivo*. The 8505C cell line was chosen in this study because it had a high tumorigenesis rate in a murine model. Animals with established flank tumors with a mean diameter of 5.0 mm were treated with daily intraperitoneal injections of placebo (*n* = 13), lower-dose dinaciclib (40 mg/kg, *n* = 13) or higher-dose dinaciclib (50 mg/kg, *n* = 7) for 12 days. Daily lower-dose dinaciclib treatment significantly retarded 8505C tumor growth by day 6 as compared to the control group (2.0 ± 0.3-fold and 2.9 ± 0.3-fold, *P* = 0.036), and the effect persisted through day 12 (2.9 ± 0.4-fold and 4.7 ± 0.4-fold, *P* = 0.007; Fig 5A). Higher-dose dinaciclib treatment significantly retarded 8505C tumor growth by day 4 (1.3 ± 0.1-fold and 2.2 ± 0.3-fold, *P* = 0.022) and the effect persisted until day 12 (2.0 ± 0.2-fold and 4.7 ± 0.4-fold, *P* < 0.001). An even lower dose of dinaciclib (30 mg/kg) had no significant effect on the growth of 8505C tumors (S4 Fig).

Daily treatment of lower-dose dinaciclib did not induce significant changes in body weight (Fig 5B). However, serial injections of higher-dose dinaciclib significantly reduced body weight when compared with control mice on day 8 (96.8 ± 2.7% and 104.2 ± 0.5%, *P* = 0.002) and the effect persisted until day 12 (95.9 ± 3.1% and 104.3 ± 0.7%, *P* = 0.003). These data reveal that the therapeutic efficacy and side effects of dinaciclib treatment were dose-dependent *in vivo*.

The molecular effects of daily dinaciclib treatment (40 mg/kg) in 8505C xenografts were evaluated (Fig 5C). Mcl-1 was decreased by 3 h and the effects persisted for 48 h. The expression of CDK1 and Aurora A was decreased between 27 h and 48 h. Cyclin B1 was decreased between 30 h and 48 h. Furthermore, Bcl-x_L_, survivin and pro-caspase-3 levels were decreased by 48 h. These data suggest that dinaciclib inhibited cell cycle progression and induced apoptosis *in vivo*.

## Discussion

Dinaciclib effectively inhibited cell proliferation in seven thyroid cancer lines of follicular cell origin, including PTC, FTC and ATC. Overall, dinaciclib has a relatively low median-effect dose *in vitro* (≤ 16.0 nM). Dinaciclib effectively represses tumor growth of ATC (8505C), suggesting that this agent has the potential for future clinical evaluations in the treatment of thyroid malignancies.

Dinaciclib caused prophase cell accumulation, which is likely one of the mechanisms of cytotoxicity in the treatment of thyroid cancer cells. The decreased levels of CDK1, cyclin B1, and Aurora A may account for this mitotic block. CDK1-cyclin B1 activity is activated in late G2 phase and the activation is required for mitotic entry. During mitosis, increasing activity of CDK1-cyclin B1 is needed for mitotic progression from prophase to prometaphase and metaphase [28]. Aurora A is a serine/threonine kinase that is also needed for G2/M transition and mitotic progression. The effects of decreased levels of CDK1, cyclin B1 and Aurora A may range from a failure in G2/M transition to mitotic arrest, depending on the magnitudes of these proteins affected. In this study, dinaciclib consistently induced mitotic block in the first mitotic phase (prophase) in three cell lines.

Besides mitotic arrest, we also noted dinaciclib accumulated cells in G2 phase in BHP7-13 cells. In this cell line, dinaciclib increased the proportion of G2/M phase cells (44.1%; Fig 2B) over that of M phase cells (18.5%; Fig 2C), indicating dinaciclib arrested cells in G2 phase. Thus, G2 phase block was likely another therapeutic mechanism of dinaciclib in BHP7-13. G2 phase arrest was not appeared in WRO82-1 and 8505C cells.

Dinaciclib decreased Mcl-1, Bcl-x_L_ and survivin levels in thyroid cancer cell lines. Mcl-1 and Bcl-x_L_ are pro-survival proteins that sequester pro-apoptotic proteins and inhibiting the apoptosis pathway [32]. Dinaciclib decreased Mcl-1 and Bcl-x_L,_ which may result in the activation of downstream executioner caspase-3 and subsequently induce apoptosis. Survivin, an inhibitor of apoptosis proteins, interacts with partners to inhibit caspase-3 [33,34]. Dinaciclib decreased survivin, which may potentiate the apoptotic effects of Mcl-1 and Bcl-x_L_ depletion.

Dinaciclib treatment inhibited 8505C tumor growth in a dose-dependent manner. The anti-tumor effect of dinaciclib is likely mediated through cell cycle inhibition and apoptosis induction given that CKD1, cyclin B1, Aurora A, Mcl-1, Bcl-x_L_, survivin and pro-caspase-3 levels were all decreased. The expression of cell cycle and apoptosis proteins was considerably altered by 48 h of dinaciclib treatment, implying that the therapeutic effect appeared following a two-dose treatment.

Paclitaxel has demonstrated therapeutic effects in patients with ATC, with a 53% response rate [35]. We evaluated the therapeutic effects of paclitaxel in mice bearing 8505C xenografts. Paclitaxel did not repress the growth of 8505C tumors after a 21-day treatment (S5 Fig). In contrary, dinaciclib was able to repress the growth of 8505C xenografts in this study. These data suggest dinaciclib may be an alternative option for patients with ATC unresponsive to paclitaxel therapy.

Among seven thyroid cancer cell lines, 8305C cells were the most resistant to 4-day treatment with dinaciclib at 25 nM. The possible mechanisms underlying the relative resistance of 8305C cells were evaluated (S6 Fig). In consistent with prior data, dinaciclib (25 nM for 24 h) accumulated cells in mitosis and inhibited mitotic progression in prophase. The proportion of cells in G2 phase arrest was also observed to increase (24% of cells being in G2/M phase and 15.9% in M phase). We also evaluated the effects of dinaciclib on the expression of proteins associated with cell cycle and apoptosis in 8305C cells (S7 Fig). As expected, treatment with dinaciclib (25 nM) for 24 h significantly decreased cyclin B1, Aurora A, Mcl-1, and Bcl-x_L_ levels. However, the magnitude of decreased survivin level was not as great in 8305C cells as that in BHP7-13, WRO82-1, and 8505C cells (S3 and S7B Figs). The failure of robust effect to decrease survivin expression may be one of the potential mechanisms accounting for dinaciclib resistance in 8305C cells.

A study of patients with acute leukemia indicated the plasma dinaciclib concentration can reach 2850.0 nM at 2 h and 92.5 nM at 6 h after initiation of a single 2-h infusion of dinaciclib (50 mg/m^2^) [36]. These plasma concentrations exceed the median-effect doses (7.1–16.0 nM) observed in our 7 thyroid cancer cell lines. We also calculated the human equivalent dose (HED) of dinaciclib based on the dosage used in mice (40 mg/kg) [37]. The HED is 121.5 mg/m^2^, which exceeds the maximally tolerated dose (50 mg/m^2^) in humans [20]. Future clinical trials are needed to clarify the efficacy of dinaciclib in the treatment of patients with thyroid cancer.

Using biomarkers to select patients who are likely to benefit from dinaciclib treatment is pivotal in clinical trials. Booher et al. reported that Mcl-1 copy number and the Mcl-1:Bcl-x_L_ mRNA ratio positively correlated with dinaciclib sensitivity in solid tumor cell lines, whereas Bcl-x_L_ mRNA level had an inverse correlation [19]. We evaluated the association between dinaciclib sensitivity and basal Mcl-1 and Bcl-x_L_ levels as well as the ratio of Mcl-1:Bcl-x_L_ in thyroid cancer cell lines (S8A Fig), and found no significant correlation (S8B Fig). However, two relatively sensitive cell lines (WRO82-1 and 8505C) had high basal levels of Mcl-1, raising the hypothesis that thyroid cancer cells that depend on Mcl-1 for survival may be susceptible to dinaciclib treatment. Analysis of more thyroid cancer cell lines with a wide range of dinaciclib sensitivity is needed to clarify this possibility. We also failed to find any association between dinaciclib sensitivity and basal survivin level in thyroid cancer cell lines (S9 Fig).

## Conclusions

The CDK inhibitor, dinaciclib, induced cytotoxicity in three major subtypes of thyroid cancer. *In vivo* analysis using 8505C xenograft tumors demonstrated the therapeutic efficacy. These data support future clinical trials studying the feasibility of dinaciclib to treat patients with refractory thyroid cancer of follicular cell origin.

## Supporting information

S1 Fig The effect of dinaciclib on mitosis in thyroid cancer cells. (A) Chromosomal appearance was evaluated in BHP7-13, WRO82-1 and 8505C cells treated with dinaciclib (25 nM) or placebo for 24 h using immunofluorescence confocal microscopy. DNA was stained with DAPI. Placebo-treated cells at metaphase (white arrows), anaphase (yellow arrows) and telophase (blue arrows) were indicated. Dinaciclib-treated cells at prophase (blue arrowheads) were demonstrated. Scale bar, 50 μm.
(TIF)

S2 Fig Effects of dinaciclib on the expression of proteins associated with the cell cycle. (A) In BHP7-13 cells, dinaciclib (25 nM) decreased CDK1 and cyclin B1 levels by 8 h and the inhibitory effects persisted for 24 h. Aurora A was transiently increased by 4 h and decreased by 6 h. (B) In WRO82-1 cells, dinaciclib (25 nM) decreased CDK1 by 8 h and the inhibitory effect persisted for 24 h. Cyclin B1 and Aurora A were transiently increased by 4 h and decreased by 6 h. (C) In 8505C cells, CDK1 was increased by 4 h and decreased by 24 h. Cyclin B1 was increased by 6 h and decreased by 24 h. Aurora A was decreased by 6 h and the inhibitory effects persisted for 24 h.
(TIF)

S3 Fig Effects of dinaciclib on the expression of proteins associated with apoptosis. (A) In BHP7-13 cells, dinaciclib (25 nM) decreased Mcl-1 level by 4 h (the effect persisting for 24 h), Bcl-x_L_ level by 16 h, and survivin level by 8 h. (B) In WRO82-1 cells, dinaciclib (25 nM) decreased Mcl-1 level by 4 h (the effect persisting for 24 h), Bcl-x_L_ level by 8 h, and survivin level by 16 h. (C) In 8505C cells, dinaciclib (25 nM) decreased Mcl-1 level by 4 h (the effect persisting for 24 h), decreased Bcl-x_L_ level by 16 h, and decreased survivin level by 8 h.
(TIF)

S4 Fig Daily intraperitoneal injections of mice with 30 mg/kg dinaciclib had no significant effect on growth of 8505C tumor xenografts over 12 days. (TIF)

S5 Fig Biweekly intraperitoneal injections of paclitaxel (0.4 mg/mouse) over a 21-day treatment period failed to repress 8505C tumor growth. (TIF)

S6 Fig Dinaciclib accumulated 8305C cells in mitosis and inhibited mitotic progression in prophase. (A) The percentage of 8305C cells in mitosis was assessed after treatment with placebo or dinaciclib (25 nM) for 24 h. Cells were stained with DAPI, and chromosome features were evaluated using immunofluorescence confocal microscopy. Mitotic index was assessed with a minimum of 941 cells counted for each condition. Dinaciclib significantly increased the proportion of 8305C cells in mitosis. (B) The distribution of cells in mitosis was determined by counting a minimum of 117 mitotic cells by confocal microscopy for each condition. All mitotic cells were found to be in prophase after treatment with dinaciclib (25 nM) for 24 h. ** *P* < 0.005 compared with vehicle-treated cells.
(TIF)

S7 Fig Dinaciclib decreased the levels of cyclin B1, Aurora A, Mcl-1, Bcl-x_L_, and survivin in 8305C cells. (A) The expression of cell-cycle and apoptosis proteins was evaluated by Western blotting in 8305C cells treated with dinaciclib (25 nM) or placebo for the indicated periods. (B) Band density was quantified using Molecular Imager VersaDoc MP 4000 system (Bio-Rad). The ratios of cyclin B1, Aurora A, Mcl-1, Bcl-x_L_, and survivin to α-tubulin were calculated. Relative expression was calculated using the control value as reference.
(TIF)

S8 Fig The association between susceptibility to dinaciclib and baseline expression of Mcl-1 and Bcl-x_L_ and the ratio of Mcl-1:Bcl-x_L_ in seven thyroid cancer cell lines. (A) Immunoblot analysis was performed to evaluate the expression of Mcl-1 and Bcl-x_L_ in seven untreated thyroid cancer cell lines. The sequence of proteins loaded was according to the Dm value of dinaciclib. (B) Band density was imaged and quantified using Molecular Imager VersaDoc MP 4000 system (Bio-Rad). The ratios of Mcl-1 and Bcl-x_L_ to α-tubulin and Mcl-1 to Bcl-x_L_ in each cell line were calculated. Relative expression was calculated using BHP7-13 value as a reference. The levels of Mcl-1 and Bcl-x_L_ and the ratio of Mcl-1:Bcl-x_L_ did not significantly correlate with dinaciclib sensitivity (Pearson correlation).
(TIF)

S9 Fig The association between susceptibility to dinaciclib and baseline expression of survivin in seven thyroid cancer cell lines. (A) Immunoblot analysis was performed to evaluate the expression of survivin in seven untreated thyroid cancer cell lines. (B) Band density was quantified. The ratios of survivin to α-tubulin in each cell line were calculated. Relative expression was calculated using the BHP7-13 value as reference. The levels of survivin did not significantly correlate with dinaciclib sensitivity (Pearson correlation).
(TIF)
