# Supplementary figures and images for: A cyclin-dependent kinase inhibitor, dinaciclib in preclinical treatment models of thyroid cancer
Source: PLoS One. 2017 Feb 16;12(2):e0172315. doi: 10.1371/journal.pone.0172315 (PMC5312924; doi:10.1371/journal.pone.0172315)

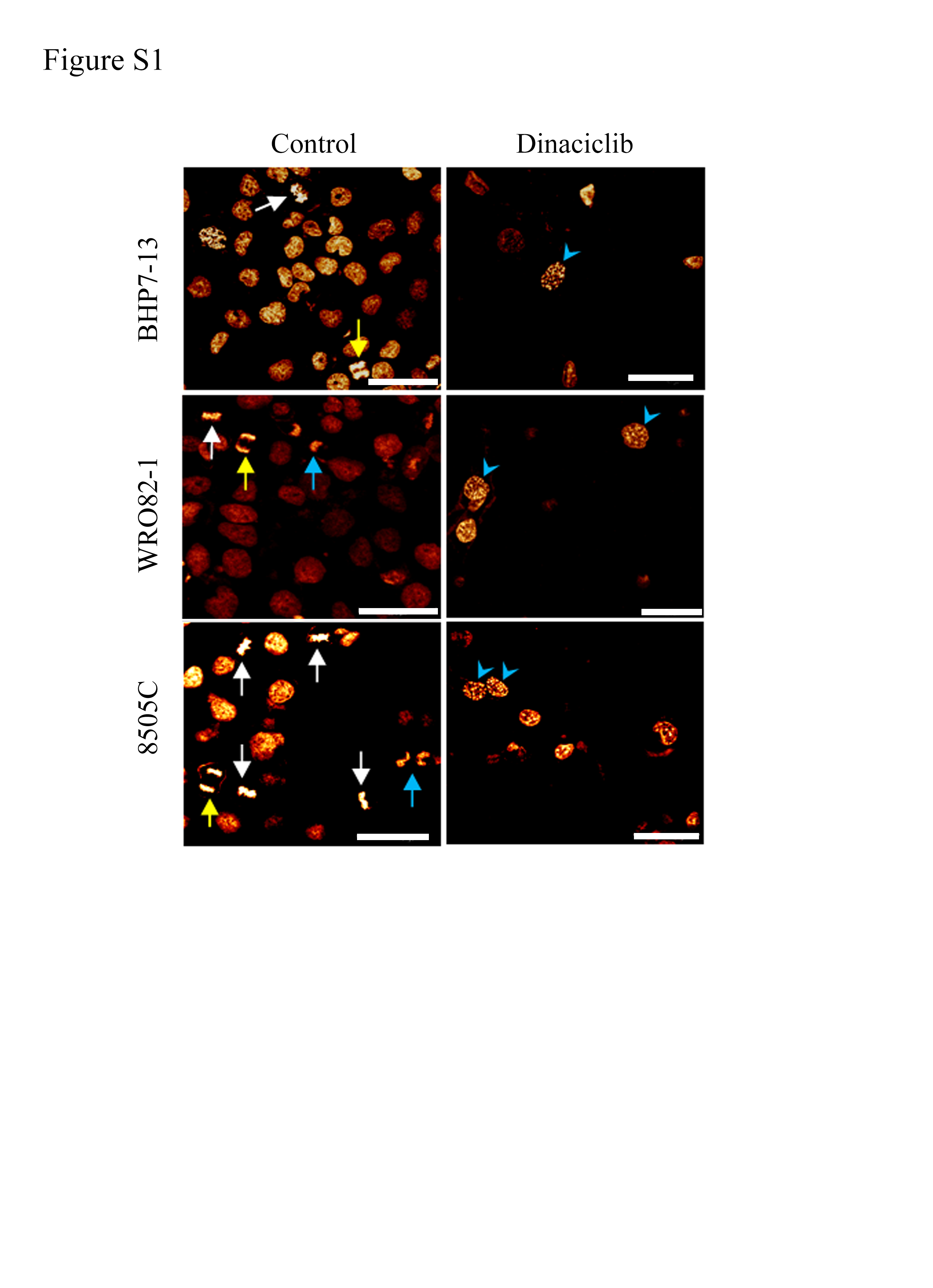

Supplement: S1 Fig — (A) Chromosomal appearance was evaluated in BHP7-13, WRO82-1 and 8505C cells treated with dinaciclib (25 nM) or placebo for 24 h using immunofluorescence confocal microscopy. DNA was stained with DAPI. Placebo-treated cells at metaphase (white arrows), anaphase (yellow arrows) and telophase (blue arrows) were indicated. Dinaciclib-treated cells at prophase (blue arrowheads) were demonstrated. Scale bar, 50 μm. (TIF) [file pone.0172315.s001.tif]

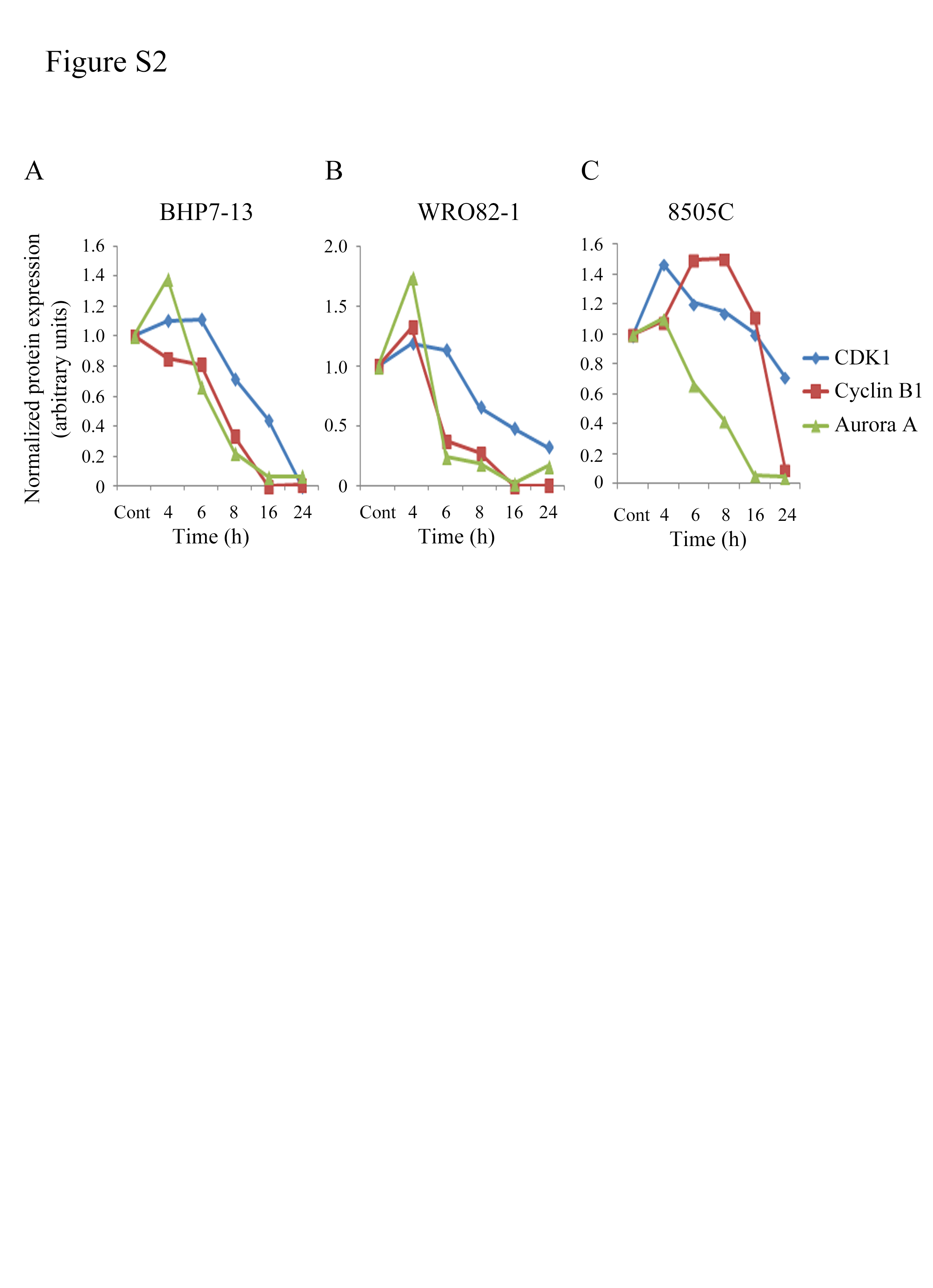

Supplement: S2 Fig — (A) In BHP7-13 cells, dinaciclib (25 nM) decreased CDK1 and cyclin B1 levels by 8 h and the inhibitory effects persisted for 24 h. Aurora A was transiently increased by 4 h and decreased by 6 h. (B) In WRO82-1 cells, dinaciclib (25 nM) decreased CDK1 by 8 h and the inhibitory effect persisted for 24 h. Cyclin B1 and Aurora A were transiently increased by 4 h and decreased by 6 h. (C) In 8505C cells, CDK1 was increased by 4 h and decreased by 24 h. Cyclin B1 was increased by 6 h and decreased by 24 h. Aurora A was decreased by 6 h and the inhibitory effects persisted for 24 h. (TIF) [file pone.0172315.s002.tif]

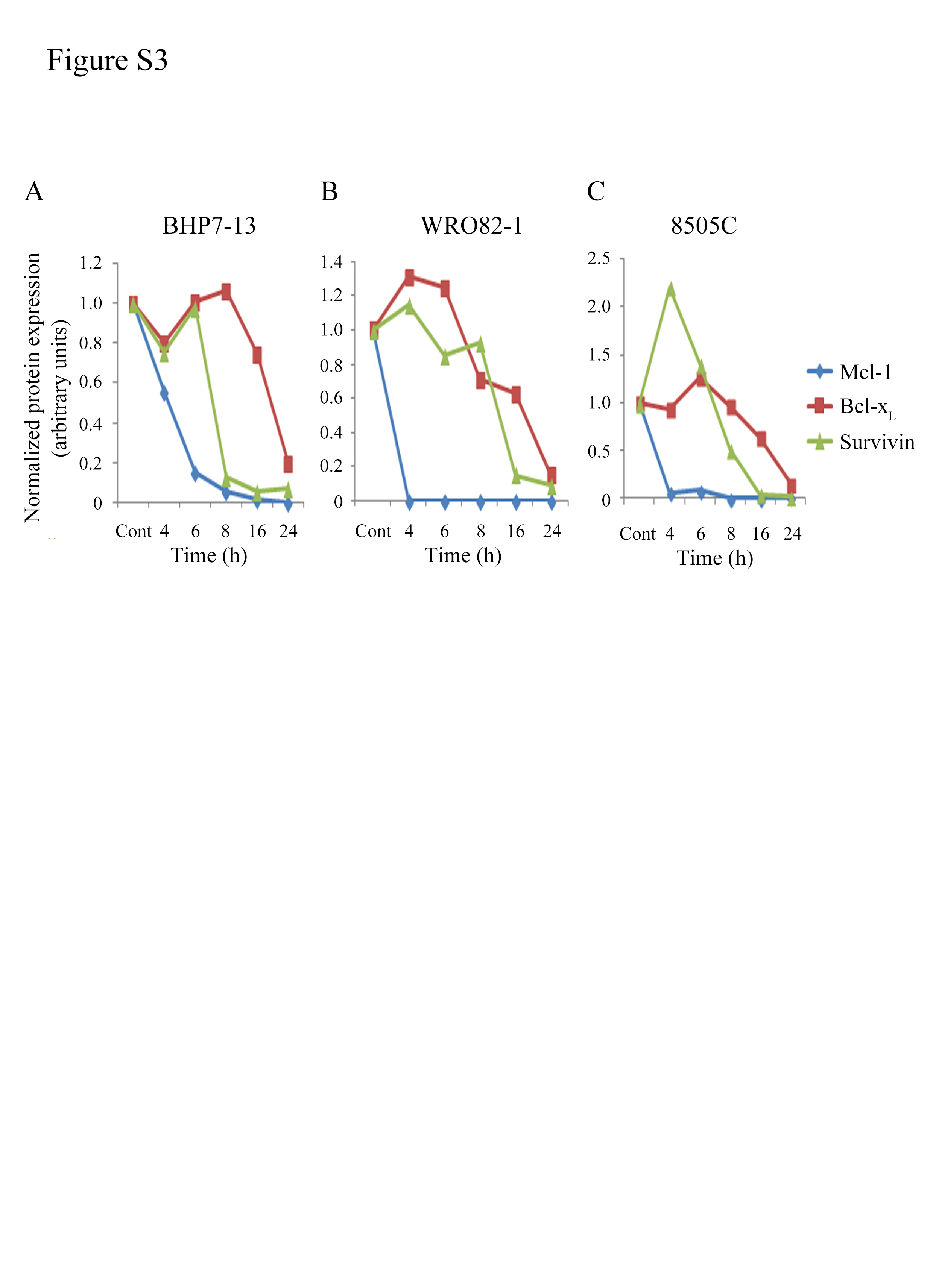

Supplement: S3 Fig — (A) In BHP7-13 cells, dinaciclib (25 nM) decreased Mcl-1 level by 4 h (the effect persisting for 24 h), Bcl-xL level by 16 h, and survivin level by 8 h. (B) In WRO82-1 cells, dinaciclib (25 nM) decreased Mcl-1 level by 4 h (the effect persisting for 24 h), Bcl-xL level by 8 h, and survivin level by 16 h. (C) In 8505C cells, dinaciclib (25 nM) decreased Mcl-1 level by 4 h (the effect persisting for 24 h), decreased Bcl-xL level by 16 h, and decreased survivin level by 8 h. (TIF) [file pone.0172315.s003.tif]

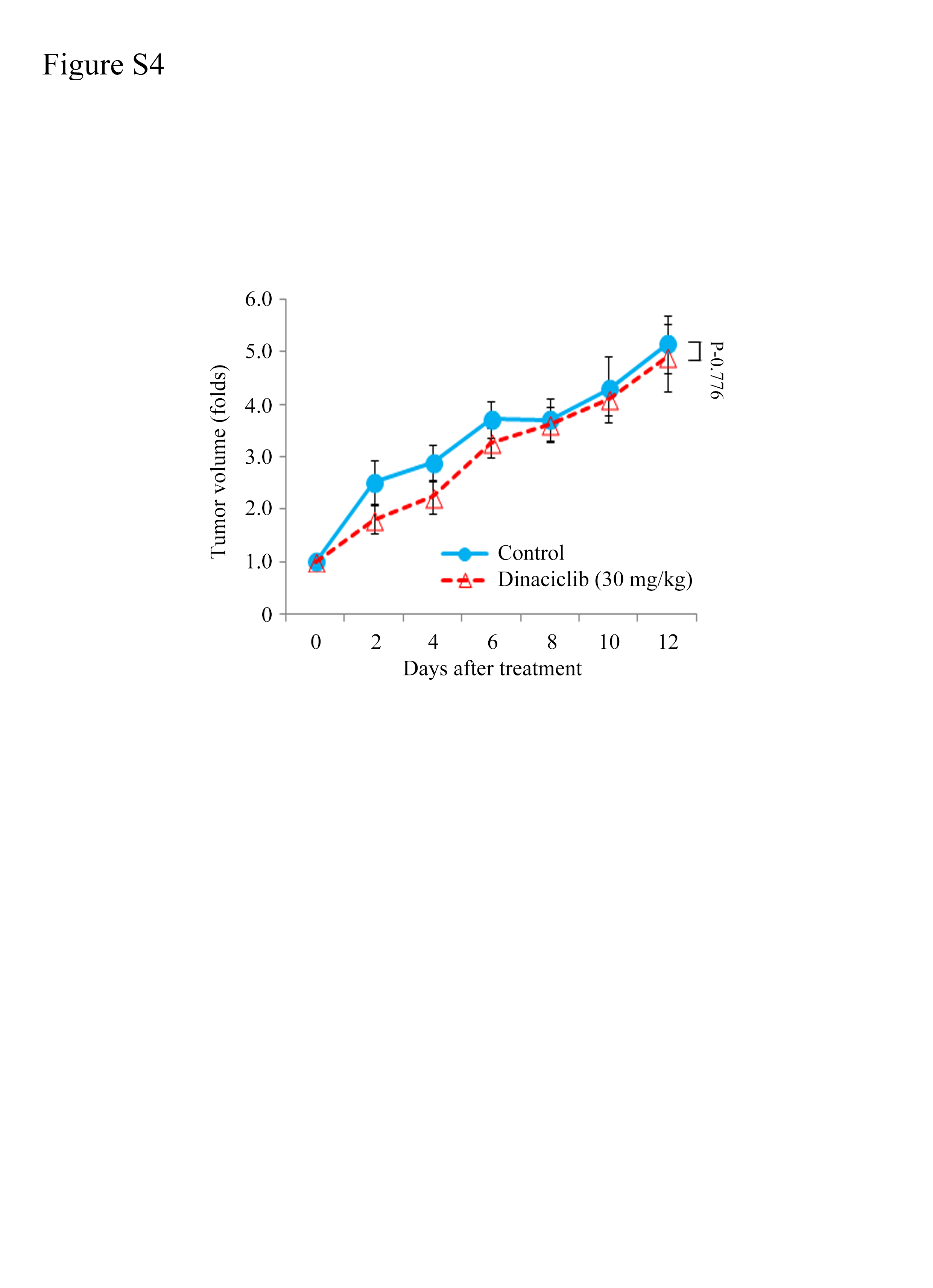

Supplement: S4 Fig — (TIF) [file pone.0172315.s004.tif]

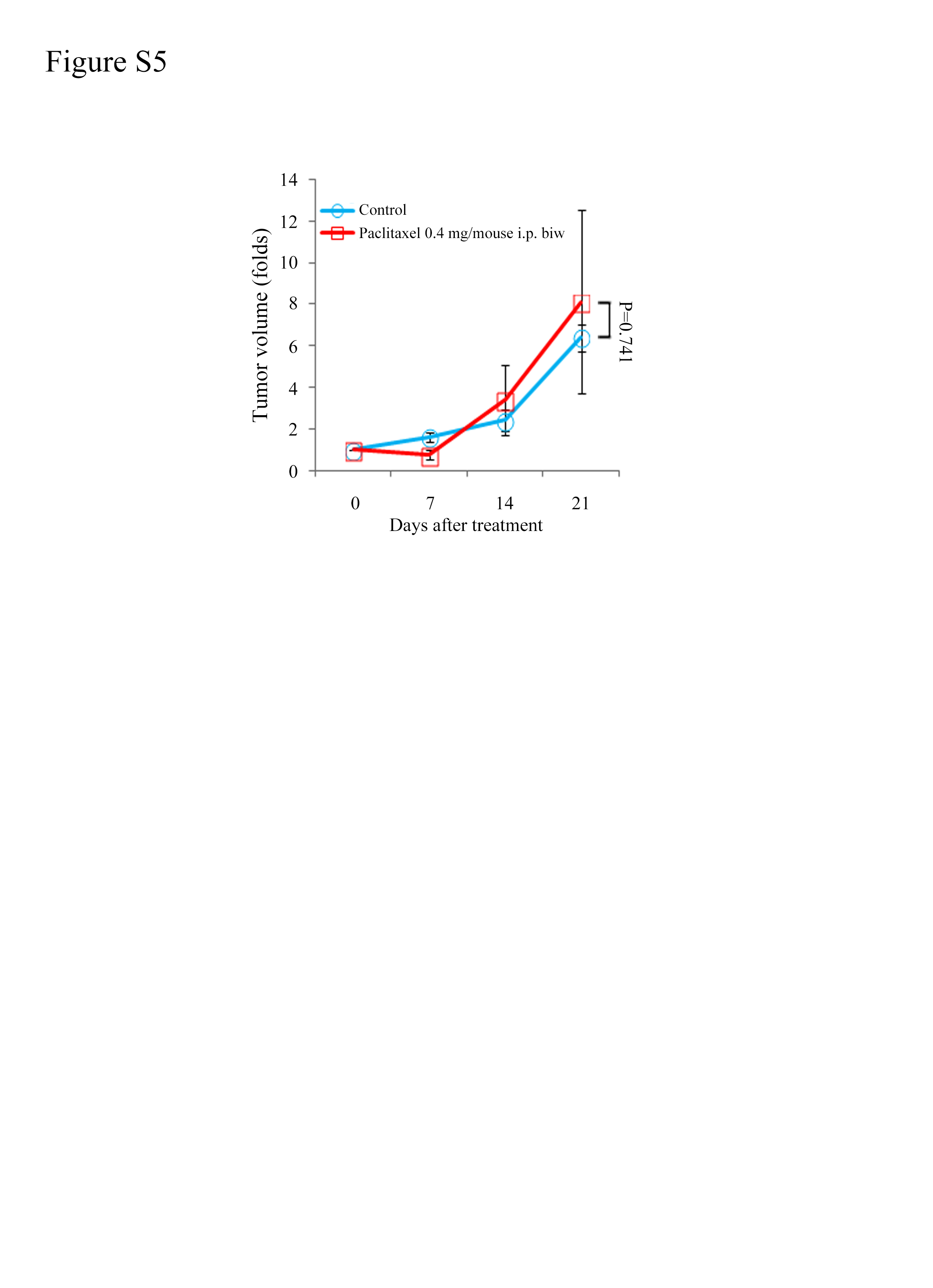

Supplement: S5 Fig — (TIF) [file pone.0172315.s005.tif]

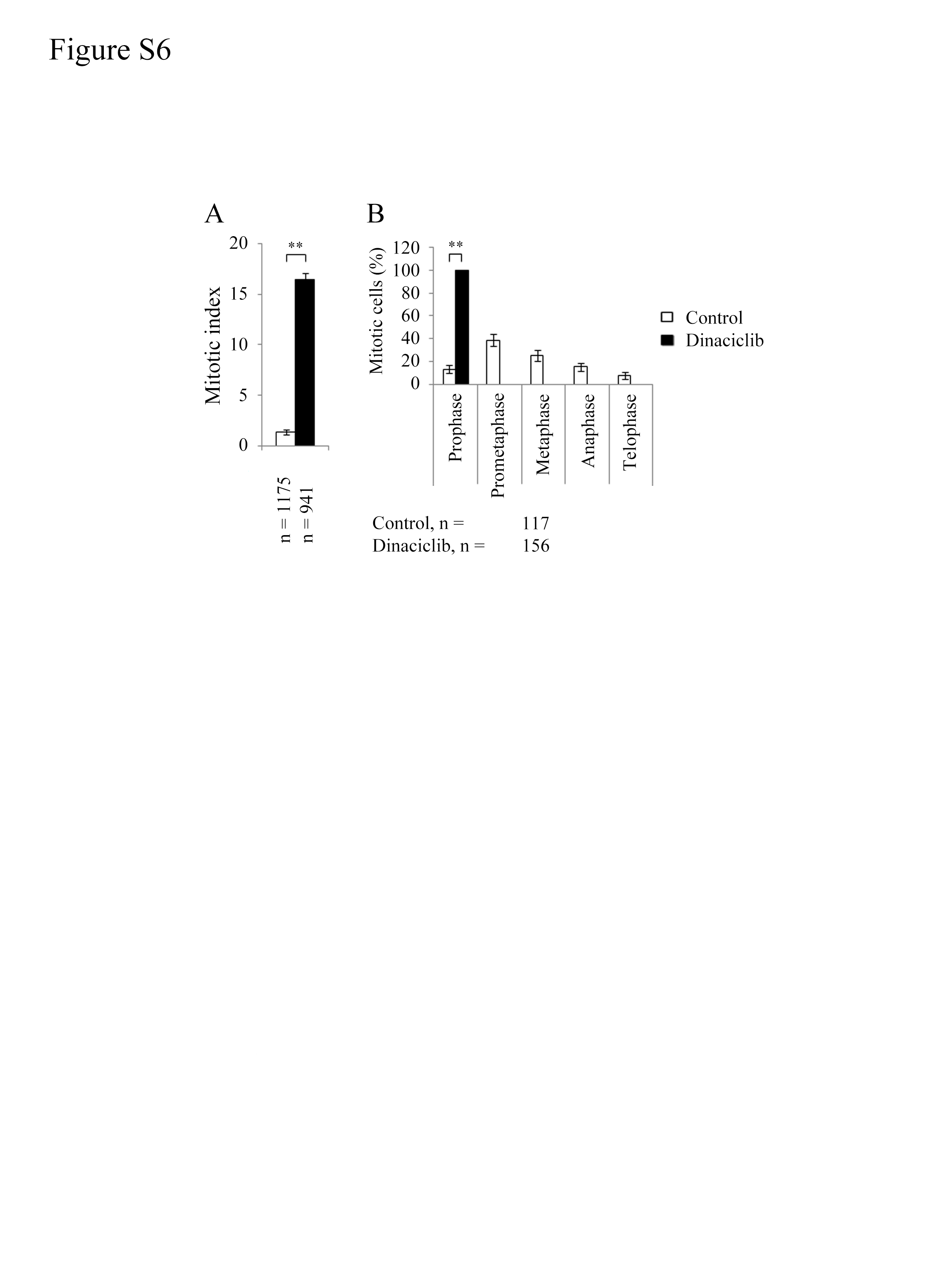

Supplement: S6 Fig — (A) The percentage of 8305C cells in mitosis was assessed after treatment with placebo or dinaciclib (25 nM) for 24 h. Cells were stained with DAPI, and chromosome features were evaluated using immunofluorescence confocal microscopy. Mitotic index was assessed with a minimum of 941 cells counted for each condition. Dinaciclib significantly increased the proportion of 8305C cells in mitosis. (B) The distribution of cells in mitosis was determined by counting a minimum of 117 mitotic cells by confocal microscopy for each condition. All mitotic cells were found to be in prophase after treatment with dinaciclib (25 nM) for 24 h. ** P < 0.005 compared with vehicle-treated cells. (TIF) [file pone.0172315.s006.tif]

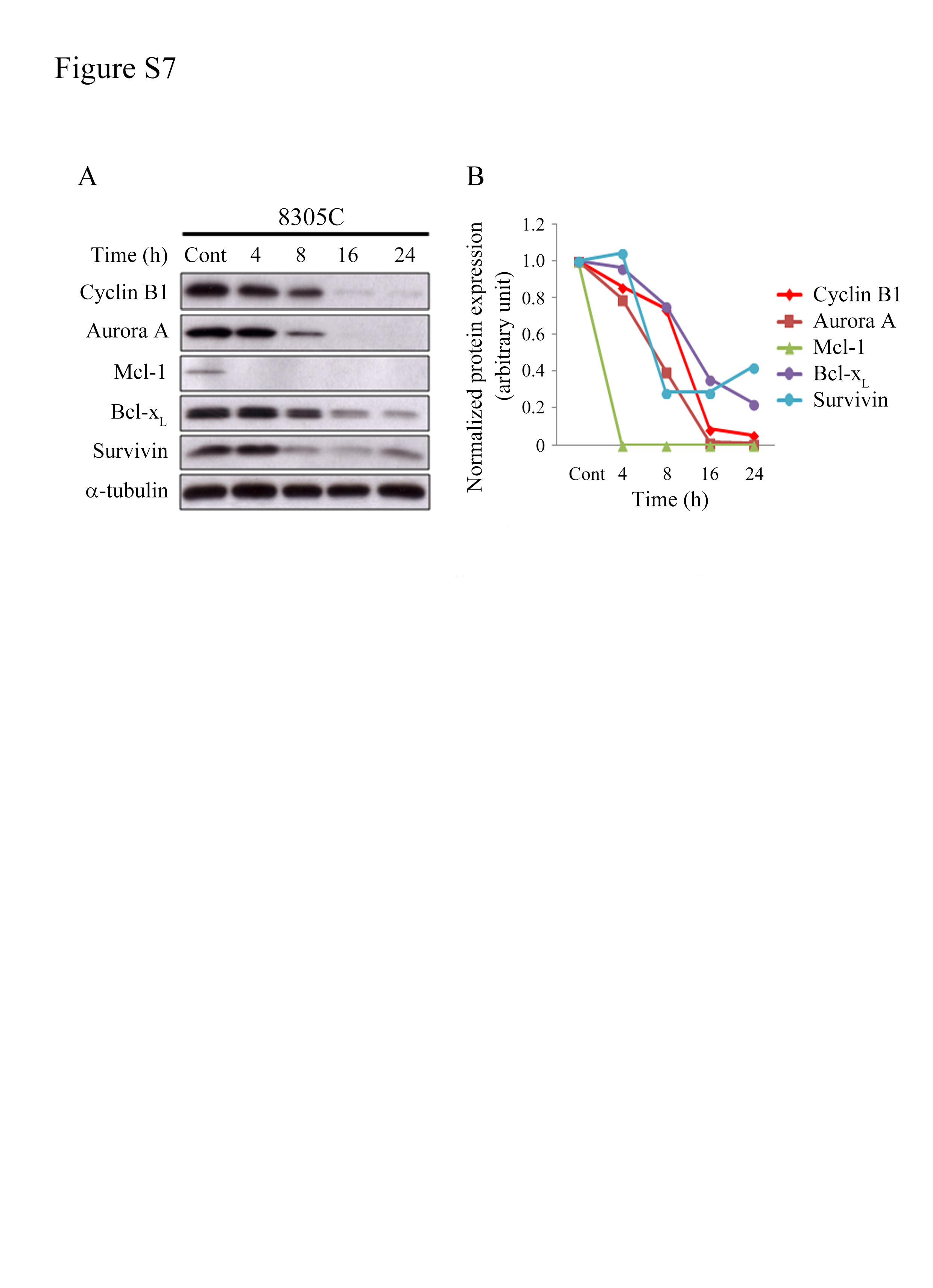

Supplement: S7 Fig — (A) The expression of cell-cycle and apoptosis proteins was evaluated by Western blotting in 8305C cells treated with dinaciclib (25 nM) or placebo for the indicated periods. (B) Band density was quantified using Molecular Imager VersaDoc MP 4000 system (Bio-Rad). The ratios of cyclin B1, Aurora A, Mcl-1, Bcl-xL, and survivin to α-tubulin were calculated. Relative expression was calculated using the control value as reference. (TIF) [file pone.0172315.s007.tif]

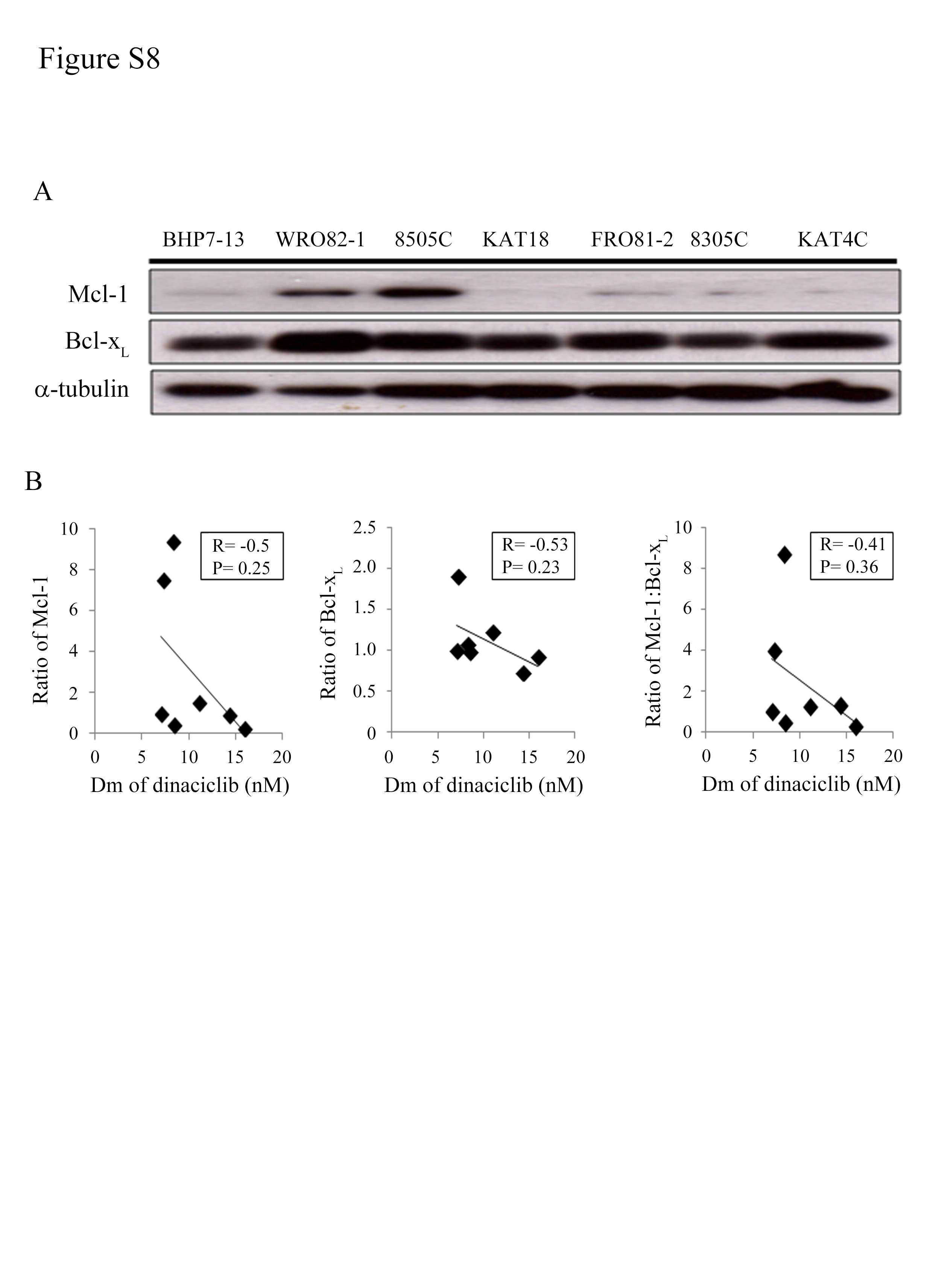

Supplement: S8 Fig — (A) Immunoblot analysis was performed to evaluate the expression of Mcl-1 and Bcl-xL in seven untreated thyroid cancer cell lines. The sequence of proteins loaded was according to the Dm value of dinaciclib. (B) Band density was imaged and quantified using Molecular Imager VersaDoc MP 4000 system (Bio-Rad). The ratios of Mcl-1 and Bcl-xL to α-tubulin and Mcl-1 to Bcl-xL in each cell line were calculated. Relative expression was calculated using BHP7-13 value as a reference. The levels of Mcl-1 and Bcl-xL and the ratio of Mcl-1:Bcl-xL did not significantly correlate with dinaciclib sensitivity (Pearson correlation). (TIF) [file pone.0172315.s008.tif]

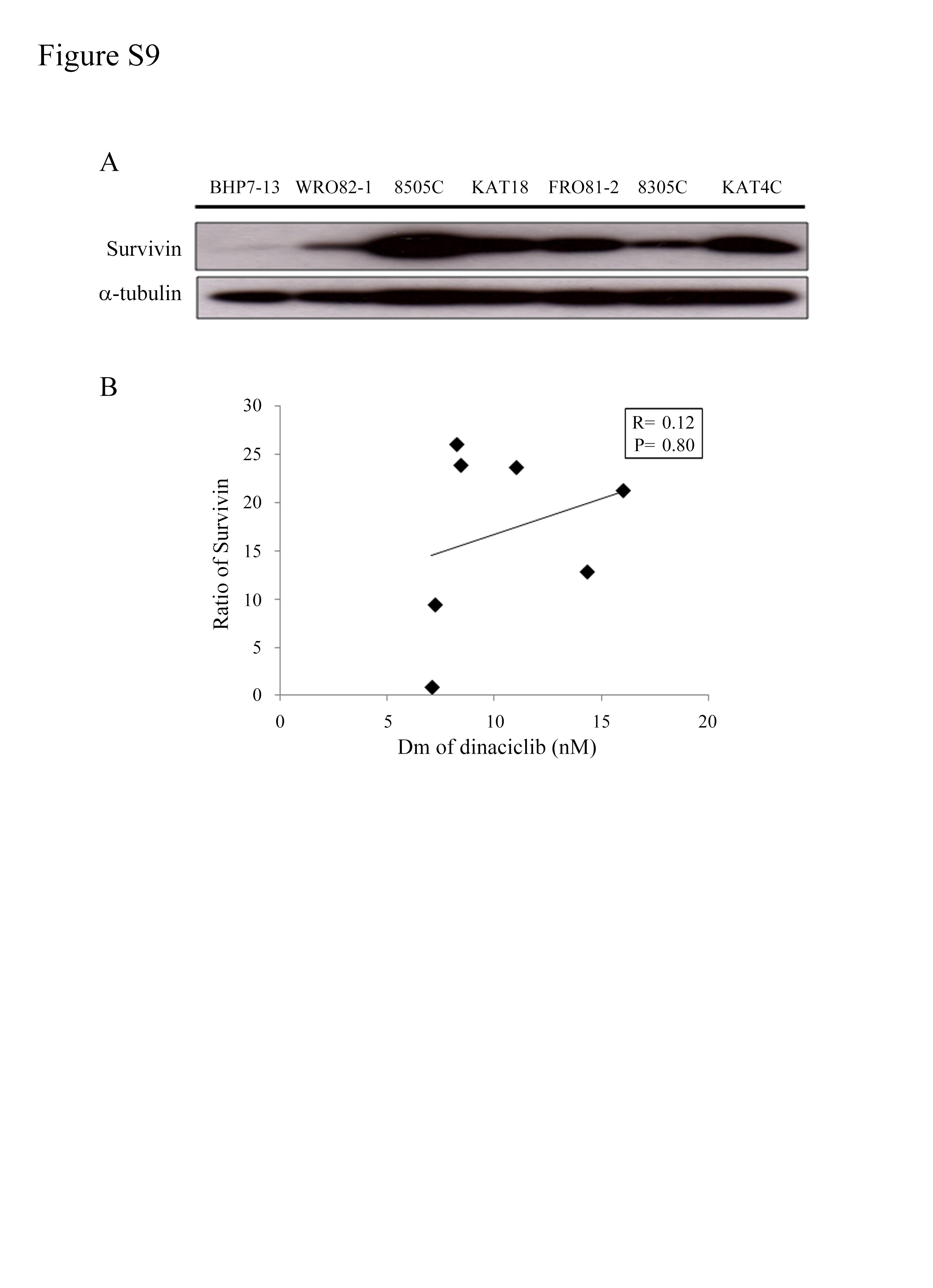

Supplement: S9 Fig — (A) Immunoblot analysis was performed to evaluate the expression of survivin in seven untreated thyroid cancer cell lines. (B) Band density was quantified. The ratios of survivin to α-tubulin in each cell line were calculated. Relative expression was calculated using the BHP7-13 value as reference. The levels of survivin did not significantly correlate with dinaciclib sensitivity (Pearson correlation). (TIF) [file pone.0172315.s009.tif]
